# Analysis of the Chemical Composition of *Inula hookeri* f. major Ling Combining UHPLC-Q-Exactive-Orbitrap MS with Feature-Based Molecular Networking and SIRIUS

**DOI:** 10.3390/molecules31152658

**Published:** 2026-07-30

**Authors:** Liangyin Shu, Wenqing Xiao, Jihui Zhao, Jieyao Ma

**Affiliations:** 1 School of Pharmaceutical Sciences, Hunan University of Medicine, Huaihua 418000, China; shuliangyin@foxmail.com (L.S.); 19573970618@163.com (W.X.); 2 College of Pharmaceutical Sciences, Zhejiang Chinese Medical University, Hangzhou 310053, China

**Keywords:** UHPLC-Q-Exactive-Orbitrap MS, *Inula hookeri* f. major Ling, tentative annotation, FBMN, SIRIUS

## Abstract

For the evaluation of the chemical composition of *Inula hookeri* f. major Ling, ultra-high-performance liquid chromatography quadrupole-Exactive Orbitrap mass spectrometry (UHPLC-Q-Exactive-Orbitrap MS) was integrated with feature-based molecular networking (FBMN) and SIRIUS. The chromatographic separation of the chemical components from *Inula hookeri* f. major Ling was conducted on a Hypersil GOLD™ aQ C_18_ (100 mm × 2.1 mm, 1.9 μm) using gradient elution, with a 0.1% formic acid aqueous solution and acetonitrile as the mobile phase. Mass spectrometric analysis was performed in both positive and negative ion modes using an electrospray ionization (ESI) source. Based on the obtained high-resolution fragment ions, FBMN was conducted to enable the clustering and visualization of structurally similar compounds. Molecular formulas were predicted and MS/MS fragment ions were interpreted with the assistance of SIRIUS. A total of 162 compounds were tentatively annotated from *Inula hookeri* f. major Ling, including terpenoids, phenylpropanoids, fatty acids, organic acids, and other types of compounds. The application of the strategy incorporating UHPLC-Q-Exactive-Orbitrap MS, FBMN, and SIRIUS enables the systematic characterization of the chemical composition of *Inula hookeri* f. major Ling.

## 1. Introduction

*Inula hookeri* f. major Ling, a *forma* of *Inula hookeri*, is an important medicinal plant recorded in the *Flora Reipublicae Popularis Sinicae* (FRPS) and traditionally used for the treatment of myoneuralgia [1]. However, to the best of our knowledge, studies regarding the chemical composition of *I. hookeri* f. major Ling have not yet been reported. This lack of phytochemical data has been a major obstacle to investigating its pharmacodynamic mechanisms and advancing its clinical application.

The high complexity of the chemical constituents in Chinese herbal extracts poses a significant challenge for traditional separation and identification methods, particularly concerning trace constituents [2]. In recent years, UHPLC-Q-Exactive-Orbitrap MS has emerged as the most commonly applied technique for characterizing the pharmacodynamic material basis of complex natural products, owing to its high resolution, sensitivity, and mass accuracy [3,4,5]. However, when dealing with massive MS datasets, manual interpretation alone exhibits extremely low efficiency. The FBMN technique can efficiently facilitate the identification of isomers and structural analogs through the visual clustering of structurally related compounds based on MS/MS fragment similarity [6,7]. Simultaneously, the SIRIUS platform excels in isotopic pattern analysis and fragment tree calculations. Consequently, the molecular formulas and detailed structural information of unknown characteristic mass spectral peaks can be intelligently predicted by combining FBMN with SIRIUS [8]. By integrating the high-throughput data acquisition of high-resolution mass spectrometry with the data-mining capabilities of FBMN and SIRIUS, an efficient and precise identification strategy for complex systems can be established [9].

In the present study, UHPLC-Q-Exactive-Orbitrap MS, FBMN and SIRIUS were combined and applied for the first time to systematically characterize the chemical composition of the *I. hookeri* f. major Ling extract. The mass spectrometric fragmentation rules of its characteristic chemical constituents—such as sesquiterpene lactones—were summarized. The chemical space network of *I. hookeri* f. major Ling was established, comprehensively revealing its material basis. To our knowledge, this represents the first study characterizing the chemical composition of *I. hookeri* f. major Ling, laying the foundation for further research, including quality control, pharmacodynamic evaluation, and lead compound development. Furthermore, this combined analytical strategy could be widely applied for the chemical characterization of other medicinal plants within the *Inula* genus, as well as other chemically complex Asteraceae species.

## 2. Results

### 2.1. Establishment of Analytical Strategy

Raw LC-MS/MS data acquired in the positive- and negative-ion modes were processed separately using MZmine. Feature detection, chromatographic deconvolution, isotope grouping, alignment, and MS/MS-spectrum association were performed to generate a feature table and an MGF file for each ionization mode.

The processed data were subsequently analyzed using feature-based molecular networking. In the molecular network, each node represents a chromatographic feature associated with an MS/MS spectrum and defined by its precursor ion and retention time. Pairwise spectral similarity was calculated from shared fragment ions and their relative intensities. An edge was retained when the cosine similarity was greater than 0.7 and at least six fragment ions were matched. Features with similar MS/MS fragmentation patterns consequently formed molecular clusters. These clusters were used to visualize spectral relationships and support the recognition of structurally related candidate groups, rather than to establish compound identities directly.

The MGF files were also analyzed using SIRIUS. SIRIUS was used to evaluate isotope patterns, predict molecular formulas, construct fragmentation trees, and rank database-derived structural candidates. The SIRIUS output was not treated as independent confirmation of a chemical structure.

Candidate annotations were manually evaluated by integrating precursor-mass accuracy, isotope patterns, ionization polarity and adduct type, diagnostic product ions and neutral losses, chromatographic retention behavior, FBMN cluster relationships, SIRIUS rankings, and previously reported mass-spectral data. A specific compound name was retained only when these lines of evidence were mutually consistent. When the available evidence could not distinguish positional or stereochemical isomers, the feature was annotated at the isomer-group or structural-class level.

FBMN clustering and chromatographic separation may indicate that features are related or chromatographically distinct, but they do not unambiguously establish the identity of individual isomers. Because authentic standards were not analyzed, the final assignments were treated as Level 3 tentative candidates.

### 2.2. Chemical Constituents in Inula hookeri f. Major Ling

**Figure 1:**
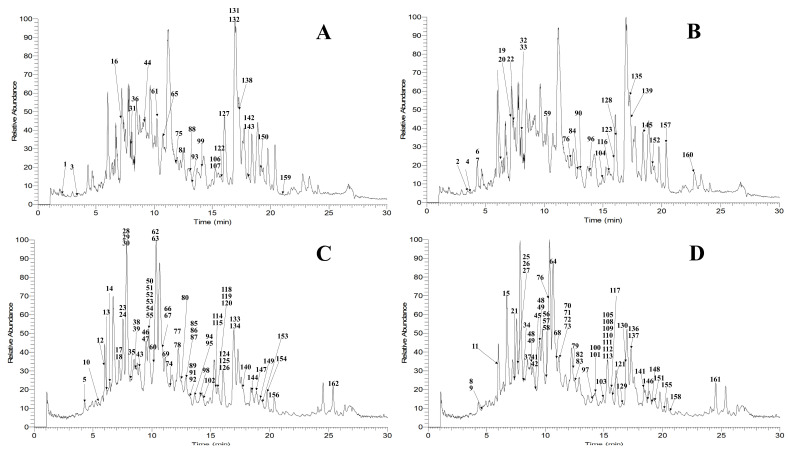
Total ion chromatograms of the *Inula hookeri* f. major extract. Panels (**A**,**B**) show the negative-ion-mode TIC with complementary sets of peak-number annotations; panels (**C,D**) show the positive-ion-mode TIC with complementary sets of peak-number annotations. The chromatograms are repeated solely to improve the legibility of the peak labels. Peak numbers correspond to Table 1. Notes: (**A**,**B**) represent the negative ion mode; (**C**,**D**) represent the positive ion mode.

The mass spectrometry data of the *Inula hookeri* f. major Ling extract were collected using UHPLC-Q-Exactive-Orbitrap MS in both positive and negative ion modes. The corresponding total ion current (TIC) chromatograms are shown in Figure 1.

Based on FBMN and SIRIUS analyses, complemented by high-resolution accurate-mass measurements, MS/MS fragmentation patterns, spectral database matching, and supporting literature data, 162 chromatographic features were assigned tentative compound annotations (Table 1). These annotations encompassed 94 terpenoids, 26 fatty acids, 15 phenylpropanoids, 4 organic acids, and 23 additional constituents. Sesquiterpene lactones were prioritized for annotation due to their status as chemotaxonomically characteristic secondary metabolites of the genus *Inula*. Phenylpropanoids were also emphasized, given the well-documented association of representatives from this class with the pharmacologically relevant bioactivities reported for *Inula* species. As no authentic reference standards were available for comparative analysis, all annotations remain tentative and are not considered structurally confirmed.

#### 2.2.1. Terpenoids

**Figure 2:**
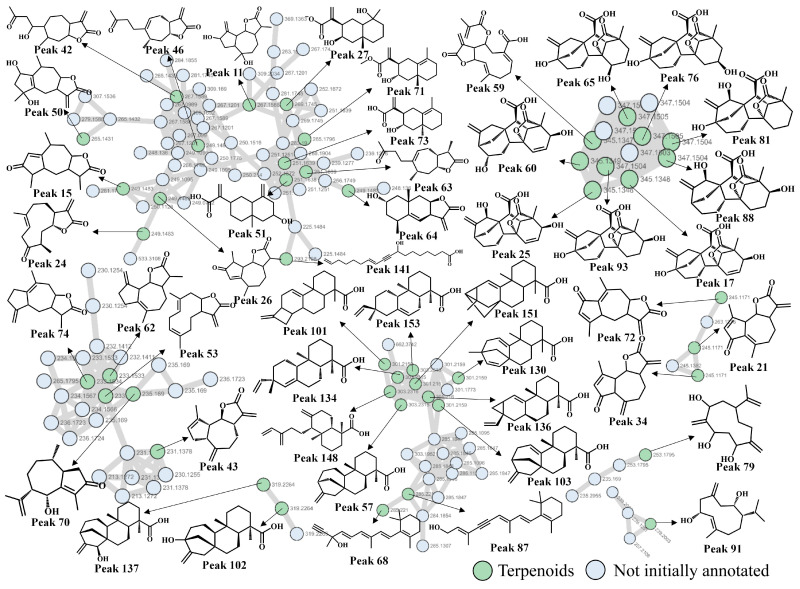
Visualized molecular clusters of terpenoids from the *Inula hookeri* f. major Ling extract.

Terpenoids constitute the main pharmacodynamic material basis of Inula species [10,11]. Based on high-resolution mass spectrometry, primarily sesquiterpene lactones and diterpenoids, were tentatively annotated from *Inula hookeri* f. major Ling in the present study. Overall, terpenoids could be detected in both positive and negative ion modes, although a higher response was observed in the positive ion mode. The common mass spectral fragmentation rules for terpenoids are as follows: the neutral loss of small molecules (H_2_O, CO_2_, CH_3_COOH, etc.), the sequential loss of side-chain substituents (methyl, methylene, acetoxy, etc., producing a fragment series with mass differences of 14 or 15 Da), characteristic lactone ring-opening cleavages, and retro-Diels-Alder (RDA) reactions. Structural isomers with similar carbon skeletons were classified, and their detailed structures were tentatively annotated using FBMN clustering analysis [12]. The visualized molecular clusters of terpenoids from the *I. hookeri* f. major Ling extract are shown in Figure 2.

Sesquiterpene lactones are considered the most important pharmacodynamic material basis of Inula species. Alantolactone (Peak 62) is a representative of this class of compounds, exhibiting a quasi-molecular ion peak at *m*/*z* 233.15340. The progressive neutral loss and side-chain alkyl cleavage occur, yielding fragment ions at *m*/*z* 215.14 and 159.11. Ivangustin (Peak 12) exhibits a precursor ion peak at *m*/*z* 249.14810, yielding a base peak fragment ion at *m*/*z* 175.11 after the loss of an oxygen-containing side chain. In the FBMN analysis, a clustering effect was observed among many sesquiterpene lactone groups. The group at *m*/*z* 249.14 includes peaks 15, 24, 26 and 46. This group undergoes selective heterolytic cleavage at the C-C bond connecting the parent skeleton and the lactone ring, yielding a base peak fragment at *m*/*z* 175.11. Based on FBMN and SIRIUS analyses, they are tentatively annotated as Trimethyl-octahydroazulenofurandione isomer, argolide, Trimethyl-octahydroazulenofurandione isomer, tomentosin. The group at *m*/*z* 229.12 includes peaks 54, 67, 85, 110 and 119. This group undergoes parent skeleton cleavage assisted by the lactone ring, yielding a key fragment ion at *m*/*z* 183.11, and then undergoes dehydrogenation and dealkylation. They are tentatively annotated as gweicurculactone and guaianolide- or cadinane-type sesquiterpene derivatives [11,12,13].

In the MS data of diterpenes, numerous highly related molecular network clusters were observed. Peaks 65, 76, 81, 88 and 93 are in the same cluster. They share the same molecular formula (C_19_H_24_O_6_), with quasi-molecular ion peaks at *m*/*z* 347.15030, 347.15042, 347.15036, 347.15039, and 347.15048, respectively. This cluster of isomers follows the same fragmentation route. The precursor ion preferentially loses one molecule of H_2_O, yielding the fragment ion at approximately *m*/*z* 329.13, and then sequentially loses H_2_O and CO_2_, yielding the key fragment ion at *m*/*z* 285.15. Based on chromatographic retention times and SIRIUS analysis, they are tentatively annotated as gibberellin candidate (C_19_H_24_O_6_). Similarly, peaks 75, 84 and 90 fall into the same cluster (experimental mass at *m*/*z* 345.13, C_19_H_22_O_6_). This cluster of isomers undergoes dehydration, yielding the fragment ion at *m*/*z* 327.12, or sequentially loses H_2_O and CO_2_, yielding the key fragment ion at *m*/*z* 283.13. They are tentatively annotated as gibberellin, and gibberellin candidate (C_19_H_22_O_6_) [14].

A series of polycyclic skeleton-containing diterpenoid acid isomers was observed in the present study. Peaks 134, 148, 153 and 156 (experimental mass at *m*/*z* 303.23, C_20_H_30_O_2_) represents a typical group of isomers. Due to the presence of a free carboxyl group in their structures, their parent skeleton undergoes preferential neutral loss of CO_2_ during MS/MS fragmentation, yielding a characteristic base peak at *m*/*z* 257.22 or other high-intensity fragment ions. Based on chromatographic retention times and fragment ion abundance ratios, they are tentatively annotated as pimaric acid, isoozic acid, isopimaric acid, and kaurenoic acid. In addition, peaks 94, 103, 130, 136 and 151 account for another group of isomers with a lower molecular weight (*m*/*z* 301.21, C_20_H_28_O_2_). During MS/MS fragmentation, they follow the aforementioned route and undergo the neutral loss of CO_2_, yielding the fragment ion at *m*/*z* 255.21. They are tentatively annotated as grandiflorenic acid and tetracyclic/pentacyclic diterpenoid carboxylic acid derivatives [14].

Other terpenoid derivatives cluster independently in the molecular network. Peak 23 features the quasi-molecular ion at approximately *m*/*z* 185.13 (C_14_H_16_) and characteristic secondary fragment ions. During MS/MS fragmentation, it preferentially loses an ethyl substituent, yielding a fragment ion at *m*/*z* 157, and then loses a methyl group, yielding the characteristic fragment ion at *m*/*z* 143. Alternatively, its parent skeleton may directly lose a methyl group, yielding the fragment ion at *m*/*z* 170. Thus, it is tentatively annotated as a chamazulene isomer.

#### 2.2.2. Phenylpropanoids

**Figure 3:**
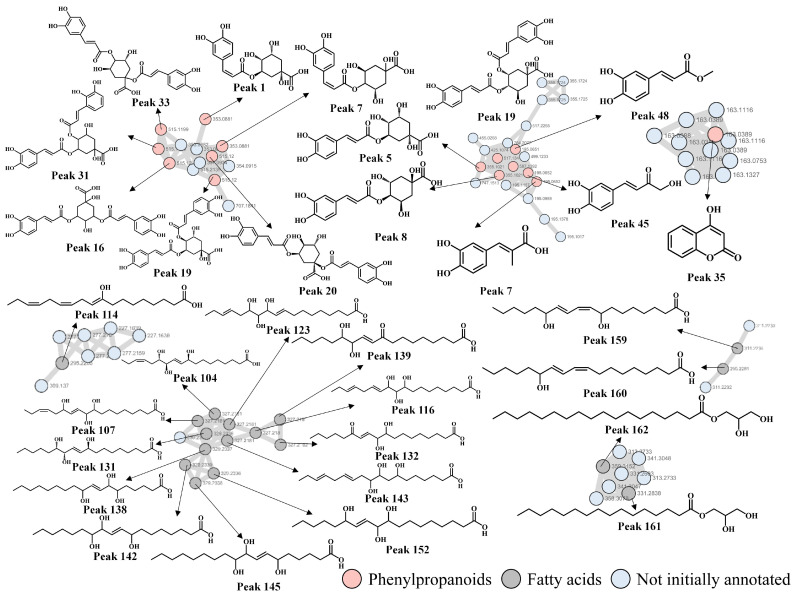
Visualized molecular clusters of phenylpropanoids and fatty acids from the *Inula hookeri* f. major Ling extract.

Phenylpropanoids (e.g., cinnamic acid derivatives, coumarins, and lignans) also contribute to the pharmacodynamic material basis of *Inula hookeri* f. major Ling. In the present study, 15 phenylpropanoid components were tentatively annotated. During MS/MS fragmentation, the preferential heterolytic cleavage of ester or glycosidic bonds is commonly observed, yielding key aromatic characteristic fragment ions (e.g., characteristic caffeoyl fragment ions at *m*/*z* 179 or 163). In addition, their parent skeletons undergo the neutral loss of small molecules (H_2_O, CO_2_, CH_3_, etc.), yielding secondary fragment ions at *m*/*z* 135 and 145. Based on the FBMN analysis, the obvious clustering of phenylpropanoid components according to their structural subtypes was observed in both the positive and negative ion modes (Figure 3) [15].

Caffeoylquinic acids exhibited an excellent response in the negative ion mode, forming highly dense molecular network clusters. Among them, dicaffeoylquinic acid isomers exhibited highly homologous fragmentation patterns. Peaks 16, 19, 20, 31, 32 and 33 share the same quasi-molecular ion at *m*/*z* 515.12 (C_25_H_24_O_12_). During MS/MS fragmentation, their ester bonds preferentially undergo heterolytic cleavage, yielding a base peak or high-abundance fragment ion representing the quinic acid skeleton at *m*/*z* 191.0555 and a dehydrated quinic acid fragment at *m*/*z* 173.0447. Simultaneously, a characteristic fragment ion at *m*/*z* 179.0342 was observed following the cleavage of the caffeoyl group. Based on chromatographic retention times and SIRIUS analysis, they are tentatively annotated as dicaffeoylquinic acid, isochlorogenic acid C, cynarine, and its isomers. The cluster of monocaffeoylquinic acid isomers (C_16_H_18_O_9_) shares the same quasi-molecular ion at approximately *m*/*z* 353.0881, along with similar characteristic fragment ions, such as the fragment ion retaining the intact caffeoyl moiety at *m*/*z* 191.05 and fragment ions generated by the neutral loss of H_2_O or CO_2_. During MS/MS fragmentation, peaks 2 and 4 preferentially undergo glycosidic bond heterolysis and lose a hexosyl group (−162 Da), yielding a caffeic acid base peak at *m*/*z* 179.0343, followed by a further neutral loss of H_2_O, yielding a fragment ion at *m*/*z* 161.0236. They are tentatively annotated as caffeic acid hexosides. Peaks 9, 45 and 48 represent a group of molecular-formula isomers sharing the same molecular formula (C_10_H_10_O_4_) and a quasi-molecular ion at approximately *m*/*z* 195.065 [M+H]^+^. Their MS/MS spectra exhibited highly similar product-ion patterns, with a base peak at *m*/*z* 163.0389 and secondary fragment ions at approximately *m*/*z* 145.0284, 135.0442, and 117.0336, indicating closely related oxygenated aromatic substructures. However, these common product ions alone cannot establish the specific connectivity or substitution pattern. Based on chromatographic behavior, FBMN relationships, and SIRIUS/database comparison, peaks 9, 45 and 48 were tentatively annotated as methyl caffeic acid, a dihydroxyphenyl-hydroxybutenone isomer, and methyl caffeate, respectively. For peak 48, the product ion at *m*/*z* 163.0389 is consistent with the neutral loss of methanol from the methyl ester, whereas no common methoxy-loss pathway was assigned to peaks 9 and 45 [15].

Coumarins and their precursors constitute an important branch of phenylpropanoids and were also tentatively annotated in the present study. Peak 35 (*m*/*z* 163.03889 [M+H]^+^) undergoes cleavage of the C–O bond, yielding an aromatic ring fragment ion, and is tentatively annotated as hydroxycoumarin isomer.

#### 2.2.3. Fatty Acids

Fatty acids exhibited responses in both the positive and negative ion modes. Their fragmentation patterns include the loss of H_2_O and CO_2_, α-cleavage, or McLafferty rearrangement along the long carbon chain, yielding a series of characteristic fragment ions of aliphatic hydrocarbons or carboxylic acids. In the present study, 26 fatty acid compounds were tentatively annotated from the *Inula hookeri* f. major Ling extract, primarily long-chain unsaturated hydroxy- and oxo-fatty acids. Based on the FBMN analysis, fatty acids with varying degrees of saturation and oxidation formed characteristic clusters (Figure 3). In the molecular network, octadecanoid polyhydroxy unsaturated fatty acids represent the largest cluster. For these compounds, differences in their MS/MS spectra mainly arise from the hydroxyl/carbonyl substitution positions and the distribution of carbon-chain double bonds [16].

Peaks 104, 107, 116, 123, 132, 139 and 143 form a cluster of isomers sharing the same quasi-molecular ion (*m*/*z* 327.21, C_18_H_32_O_5_). Their MS/MS spectra contained product ions at *m*/*z* 229.14, 211.13, and 197.11, together with ions generated by carbon-chain cleavage. Based on chromatographic retention times and SIRIUS analysis, they are tentatively annotated as malyngic acid, Trihydroxyoctadecadienoic acid isomer, trihydroxyoctadeca-dienoic acid, trihydroxyoctadeca-dienoic acid, dihydroxy-oxooctadec-enoic acid, dihydroxy-oxooctadec-enoic acid, and trihydroxyoctadeca-dienoic acid. Peaks 131, 138, 142, 145 and 152 represent another cluster of isomers (*m*/*z* 329.23, C_18_H_34_O_5_). These compounds follow the similar fragmentation rules mentioned above, namely stepwise dehydration and bond cleavage. They are tentatively annotated as pinellic acid, trihydroxy-octadecenoic acid, tianshic acid, sanleng acid, and trihydroxy-octadecenoic acid.

For unsaturated fatty acids with a low degree of oxidation, their fragment ions can more easily indicate the positions of double bonds and isolated oxygen substituents. For instance, peak 114 (*m*/*z* 295.22644 [M+H]^+^) undergoes preferential carbon chain heterolysis assisted by double bonds, yielding a characteristic fragment ion at *m*/*z* 121.1013. Based on SIRIUS analysis, it is tentatively annotated as hydroxylinolenic acid. Peak 160, located in the same cluster as peak 114, is tentatively annotated as Hode, a structurally related oxygenated C18 fatty-acid candidate. Peaks 111, 117 and 144 form a cluster of isomers (approximately *m*/*z* 293.21, C_18_H_28_O_3_). They undergo C-C bond cleavage, stably yielding characteristic fragment ions at *m*/*z* 107.08 and 81.07, and are tentatively annotated as Kot and its structural analogs.

Esterified fatty acids (monoglycerides) exhibited long chromatographic retention times due to their weak polarity. Peaks 161 and 162 showed quasi-molecular ions at *m*/*z* 331.28381 [M+H]^+^ and 359.31522 [M+H]^+^, respectively. During MS/MS fragmentation, they preferentially undergo acyl C–O bond cleavage, yielding high-abundance aliphatic hydrocarbon fragment ion peaks and consecutive fragment ions (e.g., at *m*/*z* 71.08). They are tentatively annotated as monopalmitin and monostearin.

#### 2.2.4. Organic Acids

Small-molecule organic acids are widely involved in the primary and secondary metabolic processes of plants. They are vital for maintaining plant physiological activities and the acid-base microenvironment of traditional Chinese medicine aqueous extracts. In the present study, 4 organic acid compounds were tentatively annotated, which are characterized by the presence of free carboxyl groups. During MS/MS fragmentation in the negative ion mode, they easily undergo neutral loss of CO_2_ and H_2_O. Based on their parent skeletons, they can be further classified into simple phenolic acids and aliphatic/alicyclic organic acids [17].

Phenolic acids possess an aromatic ring structure. Their fragmentation routes are characterized by the loss of CO_2_ and the cleavage of methoxy or other substituents. Peak 3 (*m*/*z* 197.04497 [M−H]^−^) undergoes the loss of methyl and methoxy groups, yielding typical fragment ions at *m*/*z* 182.02 and 166.99, and is tentatively annotated as syringic acid. Regarding phenolic acid glycoside derivatives, for instance, peak 133 (*m*/*z* 345.08099 [M+H]^+^) undergoes preferential heterolytic cleavage of the glycosidic bond, losing a glucuronic acid group and yielding an aglycone fragment ion at *m*/*z* 169.05, which further loses CO_2_, yielding a fragment ion at *m*/*z* 125.09. It is tentatively annotated as vanillic acid glucuronide [17].

Peak 6 (*m*/*z* 191.05547 [M−H]^−^, C_7_H_12_O_6_) exhibits a highly stable skeleton. During MS/MS fragmentation, the precursor ion itself constitutes the base peak. It undergoes the ring-opening of the alicyclic ring, yielding a small-molecule fragment ion at *m*/*z* 85.02. It is tentatively annotated as quinic acid, an alicyclic organic acid [17].

#### 2.2.5. Others

In addition to the aforementioned compound classes, 23 additional constituents were tentatively annotated, including polyynes, simple aromatic derivatives, nitrogen-containing compounds, and other structurally diverse constituents. Peak 49 (*m*/*z* 587.13947 [M+H]^+^, C_28_H_26_O_14_) was tentatively annotated as a taxifolin hexoside hydroxybenzoate isomer based on accurate-mass measurement, MS/MS fragmentation, FBMN relationships, and SIRIUS/database comparison. Its product-ion spectrum contained a base peak at *m*/*z* 163.0389, together with fragment ions at *m*/*z* 145.0285, 135.0442, 117.0335, and 107.0490 [11,18].

Polyynes are characteristic components with high chemotaxonomic significance for Asteraceae plants. They all exhibit a quasi-molecular ion at approximately *m*/*z* 183.116 [M+H]^+^ (C_14_H_14_). Their fragmentation route mainly involves the loss of terminal alkyl groups (methyl or ethyl) and the direct cleavage of alkyne bonds, stably yielding a series of aromatic hydrocarbon fragment ions at *m*/*z* 168.09, 155.08, and 141.06, with mass differences of 13–14 Da. Based on fragment ion abundance ratios and intelligent prediction, they are tentatively annotated as isomers, such as Dimethyl-bis(propynyl)benzene isomer, Methyl-propynyl-cyclodecapentaene isomer, 1,2-bis(but-2-ynyl)benzene, and others.

For simple aromatic derivatives, their fragmentation usually involves the loss of side-chain carbonyl and methoxy groups. Peak 10 (experimental *m*/*z* approximately 183.065, [M+H]^+^) undergoes decarbonylation (−28 Da) and demethoxylation (−31 Da), yielding a base peak at *m*/*z* 123.04, and is tentatively annotated as syringaldehyde. Peak 14 (*m*/*z* 183.06511 [M+H]^+^) undergoes dehydration and skeletal rearrangement, yielding a base peak at *m*/*z* 165.05, and is tentatively annotated as methyl-trihydroxyacetophenone isomer, a polyhydroxyacetophenone derivative.

## 3. Discussion

The characterization of the compounds in complex traditional Chinese medicine systems is often limited by the low efficiency of massive mass spectrometry data interpretation and the difficulty in distinguishing isomers. In the present study, a comprehensive strategy incorporating UHPLC-Q-Exactive-Orbitrap MS with FBMN and SIRIUS was developed and applied for the first time to systematically characterize the chemical composition of *Inula hookeri* f. major Ling. Through the in-depth integration of exact mass data from high-resolution mass spectrometry and the secondary fragment similarity clustering algorithm of FBMN, the visual dimensionality reduction of massive datasets was realized. Consequently, the identification efficiency for complex isomers (e.g., mono- and dicaffeoylquinic acids, polycyclic diterpenoids) was drastically improved. This strategy could be widely applied to the tentative annotation of complex natural products in future studies.

In the present study, a total of 162 compounds were tentatively annotated, including terpenoids (94 compounds), fatty acids (26 compounds), phenylpropanoids (15 compounds), organic acids (4 compounds), and others (23 compounds). Among them, terpenoids not only constitute the absolute majority in quantity but also form highly dense characteristic clusters in the molecular network. Special attention should be paid to sesquiterpene lactones (e.g., alantolactone, aromaticin, and various guaianolide derivatives) and diterpenoids, which are characteristic of Inula species. Terpenoid-related features represented the largest proportion of the tentative annotations obtained in this study, suggesting that terpenoids may constitute an important class of secondary metabolites in *I. hookeri* f. major. However, the number of annotated features does not represent their relative abundance or pharmacological contribution.

Previous studies have reported anti-inflammatory, cytotoxic, and other biological activities for several sesquiterpene lactones isolated from members of the genus Inula [19,20,21,22,23,24,25,26,27,28]. Some features in the present study were tentatively annotated as candidates related to these compounds, including alantolactone-, tomentosin-, and other sesquiterpene-lactone-related structures. Phenylpropanoids tentatively annotated in the extract have likewise been associated with anti-inflammatory or antioxidant activities in previous studies [29,30,31,32,33].

The prioritization of sesquiterpene lactones and phenylpropanoids reflects their established chemotaxonomic significance within the genus *Inula* and the well-documented pharmacological profiles associated with representative compounds from these classes. Nevertheless, the current qualitative LC-MS/MS profiling does not permit quantitative assessment of relative abundances, nor does it enable attribution of specific pharmacological effects or biological activities to individual constituents in *I. hookeri* f. major. Consequently, all tentative annotations should be treated as hypothesis-generating leads, requiring subsequent validation through orthogonal approaches, including analysis with authentic reference standards, targeted quantification, preparative isolation, and functional bioactivity assays.

## 4. Materials and Methods

### 4.1. Materials and Reagents

Chromatographic-grade methanol and acetonitrile were procured from Merck (Kenilworth, NJ, USA). Distilled water was sourced from Watsons Food & Beverage Co., Ltd. (Guangzhou, China). LC-MS-grade formic acid was acquired from Fisher Scientific (Waltham, MA, USA). All other reagents used were of analytical grade.

*Inula hookeri* f. major Ling was obtained from Kunming Plant Branch Biotechnology Co., Ltd., Yunnan, China, and identified by Professor Wei Cai, Hunan University of Medicine. Plant specimens are deposited in the Pharmacognosy Specimen Room, School of Pharmacy, Hunan University of Medicine (Specimen No.: HNUM20251101).

### 4.2. Sample Preparation

The powder (20 g) of dried whole herb of *Inula hookeri* f. major Ling was placed in a round-bottom flask and extracted with 70% ethanol through reflux extraction for 3 times (400 mL for each time). The extraction solutions were combined, concentrated under reduced pressure, and extracted with ethyl acetate twice. After the removal of ethyl acetate, around 5 mg of the extract was dissolved in 20 mL of methanol. After filtration, the test sample solution was obtained.

### 4.3. Liquid Chromatographic Conditions

The components in *Inula hookeri* f. major Ling were separated through a chromatographic column (Thermo Scientific Hypersil GOLD™ aQ C_18_ (Thermo Fisher Scientific, San Jose, CA, USA), 2.1 × 100 mm, 1.9 μm). The column temperature was set at 40 °C, and the flow rate was set at 0.3 mL/min. Each LC-MS analysis was interfaced with the Thermo Scientific Dionex Ultimate 3000 RS via an ESI source (Thermo Fisher Scientific Co., Ltd., Waltham, MA, USA) and performed using the Q-Exactive Focus Orbitrap MS. The mobile phase consisted of a 0.1% formic acid aqueous solution (A) and acetonitrile (B). The flow rate was set at 0.3 mL/min, and the optimized gradient elution program was as follows: from 0 to 2 min, 5–10% B; from 2 to 5 min, 10–20% B; from 5 to 10 min, 20–25% B; from 10 to 12 min, 25–55% B; from 12 to 20 min, 55–80% B; from 20 to 25 min, 80–95% B; from 25 to 26 min, 95–5% B; from 26 to 30 min, hold at 5% B, with the injection volume being 2 μL.

### 4.4. MS Conditions

Mass spectrometry analysis was conducted using the Q-Exactive Focus Orbitrap MS (Thermo Fisher Scientific, Bremen, Germany), which is equipped with a heated electrospray ionization source (HESI). Data acquisition in both positive- and negative-ion modes was performed via full-scan data-dependent MS/MS (Full-scan-DDMS2), covering a mass range of *m*/*z* 100–1500. The key parameters were as follows. The capillary voltage was set to 3.5 kV for positive-ion mode and 3.2 kV for negative-ion mode. The flow rates for sheath gas and auxiliary gas were set at 35 and 10 arb, respectively. The temperatures for capillary tube and heater were set at 320 and 350 °C, respectively. The S-lens RF level was set at 60. The stepped normalized collision energy was set at 20%, 40% and 60%, respectively. Xcalibur version 4.2 and Compound Discoverer version 3.0 (Thermo Fisher Scientific Co., Ltd., Waltham, CA, USA) were used for the collection and processing of MS data.

### 4.5. Data Analysis

The same *Inula hookeri* f. major extract was injected separately in the positive- and negative-ion modes, generating one raw dataset for each ionization mode. The positive- and negative-ion datasets were processed independently using MZmine version 4.6.1. Identical data-processing parameters were applied to the two datasets, except for the polarity and adduct settings. The protonated ion [M+H]^+^ was considered in the positive-ion workflow, whereas the deprotonated ion [M−H]^−^ was considered in the negative-ion workflow.

MS data for the tentatively annotated compound were processed using MZmine (version 4.6.1), an open-source framework for mass spectrometry data processing. During the process, mass detection was firstly performed in centroid mode, with the noise level set at 1.0 × 10^4^. The parameters for chromatogram builders were as follows. The MS level was set at 1. The ionization polarity was set separately to positive or negative according to the corresponding raw dataset. The spectrum type was set at any. The minimum consecutive scans were set at 8. The minimum intensity for consecutive scan was set at 3.0 × 10^3^. The minimum absolute peak height was set at 7.0 × 10^3^. The scan-to-scan *m*/*z* tolerance was set at 10 ppm. The Local minimum feature resolver module was used for feature deconvolution. The parameters were set as follows. The original feature list was kept. The MS/MS scan pairing was enabled. Dimension was set at retention time. The chromatographic threshold was set at 90%. The minimum search range for RT/Mobility was set at 0.05. The minimum relative peak height was set at 0%. The minimum absolute peak height was set at 7.0 × 10^3^. The minimum peak apex/edge ratio was set at 2.00. The peak duration was set at 0.00~1.00 min. The minimum scans were set at 8. The *m*/*z* and retention time tolerance for isotopic peak grouper were set at 3.5 ppm and 0.01 min, respectively. The parameters for the join aligner were as follows. The *m*/*z* tolerance was set at 5 ppm. The weight was set at 3. The retention time tolerance was set to 0.10 min. Finally, the chromatographic peaks with retention time in the range of 0.50 to 30.00 min were kept after the screening of feature list rows filter. After MZmine processing, MS/MS spectrum summary file (.mgf) and feature quantification table (.txt) were obtained and submitted to FBMN for analysis. The mass tolerances of precursor ions and fragment ions were all set at 0.02 Da. A molecular network was then generated, and its edges were filtered to retain only those with a cosine score above 0.7 and more than 6 matched peaks.

The .mgf file was imported into SIRIUS (version 6.3.3), and the “Compute All” process was executed. The global configuration was as follows. The instrument was Orbitrap. The MS/MS mass accuracy was set at 5 ppm. The fallback adducts were [M−H]^−^ and [M+H]^+^. All databases were selected. Spectral matching was enabled. The precursor deviation was set at 10 ppm. For the molecular formula identification module, the SIRIUS and ZODIAC algorithms were enabled. The “De novo + bottom up (recommended)” strategy was selected. The “Perform de novo below *m*/*z*” was set at 1000, with the element filter enabled. The allowed elements included H, C, N and O (Automatic detection was disabled). In addition, the Predict, Score threshold, Search DBs and MSNovelist modules were enabled, with the option of “pubchem as fallback” disabled. Features with similar precursor ions and fragmentation patterns were grouped as candidate isomers, whereas distinct retention times indicated chromatographically resolved features. Because authentic standards were not analyzed, the exact positional and stereochemical identities of these features remain tentative. Authentic reference standards were not analyzed in the present study. Therefore, the compound assignments were classified as tentatively annotated.

Solvent blanks were acquired under the corresponding LC-MS conditions. Sample and blank features were compared according to their precursor *m*/*z* and retention time. Features whose intensity in the sample was less than 1000% of the intensity of the corresponding blank feature (sample-to-blank intensity ratio < 10) were considered blank-related and were removed by manual review. Features not detected in the solvent blank were retained. A pooled QC sample was not prepared because the study involved qualitative profiling of a single plant extract, with one injection performed in each ionization mode. Consequently, QC-based repeatability assessment and coefficient-of-variation filtering were not performed. Isotopic features were grouped using an *m*/*z* tolerance of 3.5 ppm and an RT tolerance of 0.01 min. Only [M+H]^+^ and [M−H]^−^ were considered to be the principal adducts in the positive- and negative-ion workflows, respectively. Isotopic peaks and redundant ion forms were not treated as independent compound annotations.

## 5. Conclusions

In summary, this study not only comprehensively fills the research gap in the chemical constituents of *Inula hookeri* f. major Ling, provides a preliminary chemical profile, but also clarifies the characteristics of terpene-dominated substances at the molecular level. Based on this chemical profiling, future research may integrate in vivo absorbed component analysis and network pharmacology approaches to conduct targeted pharmacological validation and mechanistic exploration of anti-inflammatory, anti-tumor and other bioactivities. The present study provides a preliminary chemical profile of *I. hookeri* f. major and identifies several compound classes that may warrant further investigation. However, the individual annotations and their proposed biological relevance require confirmation through authentic standards, quantitative analysis, compound isolation, and pharmacological evaluation. These findings may support future studies on quality evaluation and bioactive-constituent discovery but do not, by themselves, establish therapeutic efficacy or clinical applicability.

## Figures and Tables

**Table 1:** Tentative annotation of chemical components in *Inula hookeri* f. major Ling by UHPLC-Q-Exactive-Orbitrap MS.

Peak	t_R_ (min)	Theoretical Mass (*m*/*z*)	Experimental Mass (*m*/*z*)	Error (ppm)	Formula	MS/MS Fragment (−)	MS/MS Fragment (+)	Tentatively Annotated
1	2.01	353.08780	353.08813	4.026	C_16_H_18_O_9_	MS[353]: 191.0555(100), 179.0344(2)		Caffeoylquinic acid isomer
2	3.45	341.08780	341.08810	4.080	C_15_H_18_O_9_	MS[341]: 179.03435(100), 161.02367(60), 135.04430(58)		Caffeic acid-hexoside isomers
3	3.51	197.04554	197.04497	2.640	C_9_H_10_O_5_	MS[197]: 182.02147(100), 166.99805(31)		Syringic acid
4	3.79	341.08780	341.08807	3.992	C_15_H_18_O_9_	MS[341]: 179.03429(100), 161.02362(46), 135.04414(62)		Caffeic acid-hexoside isomers
5	4.29	355.10235	355.10220	−0.447	C_16_H_18_O_9_	MS[353]: 191.0555(100), 173.0448(1), 179.0340(1)	MS[355]: 163.0390(100), 145.0284(7), 135.0443(3), 117.0338(1)	Chlorogenic acid isomers
6	4.31	191.05611	191.05547	2.384	C_7_H_12_O_6_	MS[191]: 191.05553(100), 85.02824(12)		Quinic acid
7	4.38	353.08780	353.08810	3.941	C_16_H_18_O_9_	MS[353]: 191.05551(100), 179.03413(2), 173.04498(1), 161.02359(2), 135.04422(1), 127.03892(1)		Chlorogenic acid isomers
8	4.65	355.10235	355.10208	−0.784	C_16_H_18_O_9_		MS[355]: 163.0390(100), 145.0285(11), 135.0442(6), 117.0336(2), 107.0494(1)	Caffeoylquinic acid isomer
9	4.70	195.06518	195.06516	−0.130	C_10_H_10_O_4_		MS[195]: 163.0389(100), 145.0284(18), 153.0545(1), 135.0442(13), 117.0337(3), 107.0495(2)	Methyl caffeic acid
10	5.64	183.06518	183.06508	−0.575	C_9_H_10_O_4_		MS[183]: 140.0468(30), 123.0443(100), 95.0496(88), 155.0704(21), 123.0806(11), 99.0445(10)	Syringaldehyde
11	5.97	267.15908	267.15881	−1.032	C_15_H_22_O_4_		MS[267]: 231.1380(57), 203.1431(68), 185.1326(100), 159.1170(32), 145.1012(94), 119.0857(58), 157.1014(90), 131.0857(42), 105.0703(54)	Dihydroxy-dimethyl-methylidene-octahydroazulenofuranone isomer
12	6.07	249.14852	249.14810	−1.690	C_15_H_20_O_3_		MS[249]: 175.11186(100), 203.14348(34), 185.13234(33), 143.08603(10)	Ivangustin
13	6.16	197.11722	197.11713	−0.461	C_11_H_16_O_3_		MS[197]: 179.1067(100), 135.1169(56), 161.0961(34), 133.1013(49), 107.0859(50), 93.0704(15)	Hydroxypentyl-benzenediol isomer
14	6.59	183.06518	183.06511	−0.411	C_9_H_10_O_4_		MS[183]: 165.0547(100), 137.0598(47), 109.0652(6), 141.0547(25), 123.0443(8), 95.0497(7)	Methyl-trihydroxyacetophenone isomer
15	6.69	249.14852	249.14827	−1.008	C_15_H_20_O_3_		MS[249]: 231.1378(12), 203.1437(25), 185.1324(21), 175.1118(100), 157.1014(18), 173.0963(31)	Trimethyl-octahydroazulenofurandione isomer
16	7.15	515.11949	515.12024	3.567	C_25_H_24_O_12_	MS[515]: 191.0556(58), 173.0449(100), 179.0344(99), 135.0442(27), 161.0237(19)		Dicaffeoyl quinic acid
17	7.20	347.18530	347.18506	−0.692	C_20_H_26_O_5_		MS[347]: 283.1691(23), 285.1848(100), 267.1743(76), 107.0860(12), 119.0858(10), 177.0909(10), 211.1115(9)	Gibberellin candidate (C_20_H_26_O_5_)
18	7.20	329.17473	329.17444	−0.898	C_20_H_24_O_4_		MS[329]: 285.1848(43), 267.1743(100), 239.1798(9), 249.1641(4), 183.1170(9)	Vernoflexin
19	7.30	515.11949	515.11993	2.965	C_25_H_24_O_12_	MS[515]: 191.0555(100), 173.0449(5)	MS[517]: 163.0390(100), 145.0285(11), 135.0442(6), 117.0338(2)	Isochlorogenic acid C
20	7.33	515.11949	515.11993	2.965	C_25_H_24_O_12_	MS[515]: 191.0556(100), 179.0342(7), 135.0442(2), 173.0447(3)		Cynarine
21	7.34	245.11722	245.11714	−0.330	C_15_H_16_O_3_		MS[245]: 227.1068(31), 199.1118(100), 181.1011(27), 171.1167(37), 173.0967(13)	Dehydroleucodine
22	7.37	427.17622	427.17422	−2.129	C_24_H_28_O_7_	MS[427]: 73.02814(46), 85.02821(94), 87.00763(15), 191.01968(13)		1-Isomangostin hydrate
23	7.55	185.13247	185.13228	−1.065	C_14_H_16_		MS[185]: 170.1091(38), 143.0856(50), 157.1012(61), 156.0933(19), 142.0779(17), 129.0702(17)	Chamazulene isomers
24	7.55	249.14852	249.14821	−1.248	C_15_H_20_O_3_		MS[249]: 203.1432(36), 185.1325(51), 175.1118(78), 157.1012(100), 131.0857(92), 133.1013(75)	Argolide
25	7.72	347.18530	347.18512	−0.520	C_20_H_26_O_5_		MS[347]: 285.1848(100), 267.1743(73), 239.1431(10), 211.1479(9), 183.1172(10)	Gibberellin candidate (C_20_H_26_O_5_)
26	7.74	249.14852	249.14830	−0.887	C_15_H_20_O_3_		MS[249]: 203.1429(26), 185.1323(18), 175.1118(100), 157.1012(36), 161.0960(13), 133.1013(22), 157.1012(36)	Trimethyl-octahydroazulenofurandione isomer
27	7.75	269.17473	269.17447	−0.987	C_15_H_24_O_4_		MS[269]: 233.1538(34), 205.1588(68), 187.1483(100), 85.0654(12), 159.1168(72), 133.1013(17)	8beta-Hydroxy ilicate
28	7.84	267.15908	267.15884	−0.920	C_15_H_22_O_4_		MS[267]: 231.1380(78), 203.1431(86), 185.1325(100), 159.1168(88), 133.1012(46), 145.1013(49), 157.1013(47)	Dihydroxy-dimethyl-methylidene-octahydroazulenofuranone isomer
29	7.84	247.13287	247.13266	−0.854	C_15_H_18_O_3_		MS[247]: 201.1276(53), 183.1169(100), 157.1012(75), 131.0855(90), 81.0705(33), 159.1169(63), 143.0857(56)	Methyl-dimethylidene-hexahydrocyclodecafurandione isomer
30	7.86	183.11682	183.11671	−0.639	C_14_H_14_		MS[183]: 168.0934(66), 155.0856(45), 153.0699(20), 154.0774(6), 141.0699(19)	Dimethyl-bis(propynyl)benzene isomer
31	7.98	515.11949	515.11981	2.723	C_25_H_24_O_12_	MS[515]: 191.0555(35), 173.0448(100), 179.0343(73), 135.0442(18)		Dicaffeoylquinic acid
32	8.06	515.11949	515.11993	2.965	C_25_H_24_O_12_	MS[515]: 191.0557(41), 173.0447(100), 179.0343(71), 135.0443(16)		Dicaffeoylquinic acid
33	8.10	515.11949	515.12000	3.101	C_25_H_24_O_12_	MS[515]: 173.04472(100), 179.03435(71), 191.05574(41), 135.04430(16), 353.09167(3)		4,5-Dicaffeoylquinic acid
34	8.11	245.11722	245.11716	−0.248	C_15_H_16_O_3_		MS[245]: 227.1075(23), 199.1118(100), 181.1008(41), 173.0960(28), 171.1168(37), 171.0803(18)	Methyl-dimethylidene-hexahydroazulenofurandione isomer
35	8.30	163.03897	163.03889	−0.494	C_9_H_6_O_3_		MS[163]: 145.0284(15), 135.0441(34), 133.0285(11), 117.0337(9), 107.0495(3), 89.0391(5)	Hydroxycoumarin isomer
36	8.47	363.18131	363.18167	4.006	C_20_H_28_O_6_	MS[363]: 319.1918(69), 317.1762(100), 301.1814(4), 275.2021(2), 273.2867(4)		Dihydroxy-dimethyl-methylidenetetracyclopentadecane dicarboxylic acid isomer
37	8.48	329.17473	329.17450	−0.716	C_20_H_24_O_4_		MS[329]: 285.1847(38), 267.1743(100), 239.1794(13), 239.1425(9), 211.1478(11)	Dimethyl-methylidene-dioxatricyclononadecatrienedione isomer
38	8.61	247.13287	247.13240	−1.906	C_15_H_18_O_3_		MS[247]: 201.1275(28), 183.1168(28), 173.0961(73), 157.1013(18), 131.0857(100), 105.0701(12), 143.0859(14)	Thieleanin
39	8.69	149.09609	149.09605	−0.279	C_10_H_12_O		MS[149]: 121.0650(94), 93.0703(10), 121.1012(8), 109.0651(10), 107.0859(8)	Tetrahydronaphthol isomer
40	8.72	261.14852	261.14835	−0.655	C_16_H_20_O_3_		MS[261]: 229.1218(100), 211.1116(13), 201.1278(47), 183.1169(89), 173.1324(10), 150.1040(63), 137.0964(46)	Methyl dimethyl-oxo-tetrahydroazulenylpropenoate isomer
41	8.87	379.21151	379.21213	1.621	C_21_H_30_O_6_		MS[379]: 144.10205(100), 185.13269(60), 157.10104(55), 143.08554(44)	Acetyl-isobutyrylvernolide isomer
42	8.90	267.15908	267.15881	−1.032	C_15_H_22_O_4_		MS[267]: 231.1380(57), 203.1431(68), 185.1326(100), 157.1170(32), 145.1012(94), 119.0857(58), 93.0703(42)	Hydroxy-oxobutyl-methyl-methylidene-hexahydrocycloheptafuranone isomer
43	8.98	231.13795	231.13780	−0.667	C_15_H_18_O_2_		MS[231]: 185.1326(100), 157.1012(61), 142.0777(7), 158.1090(18), 143.0856(20), 129.0700(4)	Isodehydrocostus Lactone
44	9.15	351.21769	351.21799	3.956	C_20_H_32_O_5_	MS[351]: 59.01241(3), 279.03064(2), 321.20755(100), 351.21841(82)		Sterebin B
45	9.22	195.06518	195.06520	0.075	C_10_H_10_O_4_		MS[195]: 163.0389(100), 145.0285(20), 135.0442(13), 117.0336(4), 107.0494(2)	Dihydroxyphenyl-hydroxybutenone isomer
46	9.47	249.14852	249.14824	−1.128	C_15_H_20_O_3_		MS[249]: 231.1381(84), 203.1432(100), 213.1277(42), 185.1325(80), 175.1118(63), 157.1011(52), 159.1167(28)	Tomentosin
47	9.53	303.23185	303.23157	−0.946	C_20_H_30_O_2_		MS[303]: 285.2215(63), 111.0807(52), 95.0860(78), 121.0648(51), 109.1015(82), 131.0856(42), 105.0703(54)	Hydroxy-dimethyl-methylidenetetracyclohexadecane carbaldehyde isomer
48	9.64	195.06518	195.06511	−0.386	C_10_H_10_O_4_		MS[195]: 163.0389(100), 145.0284(17), 135.0441(12), 117.0337(4), 95.0859(4)	Methyl Caffeate
49	9.64	587.13953	587.13947	−0.105	C_28_H_26_O_14_		MS[587]: 163.0389(100), 145.0285(13), 135.0442(9), 117.0335(2), 107.0490(1)	Taxifolin hexoside hydroxybenzoate isomer
50	9.72	265.14343	265.14304	−1.492	C_15_H_20_O_4_		MS[265]: 229.1220(88), 247.1330(34), 219.1378(22), 201.1273(93), 183.1167(43), 173.1324(39), 189.0908(86), 187.1118(56), 159.1168(100), 119.0858(47)	Dihydroxy-dimethyl-methylidene-hexahydroazulenofuranone isomer
51	9.72	251.16417	251.16377	−1.597	C_15_H_22_O_3_		MS[251]: 233.1539(23), 205.1588(35), 187.1480(53), 177.1274(23), 175.1116(24), 159.1168(100), 147.1169(45), 133.1012(32)	Hydroxy-methyl-methylidene-decahydronaphthalenyl acrylic acid isomer
52	9.72	247.13287	247.13252	−1.420	C_15_H_18_O_3_		MS[247]: 187.1119(23), 159.1168(34), 85.0653(100), 57.0707(88), 69.0342(26), 229.1224(35), 102.1274(56), 183.1167(49)	Dimethyl-methylidene-hexahydroazulenofurandione isomer
53	9.72	233.15360	233.15335	−1.100	C_15_H_20_O_2_		MS[233]: 187.1481(27), 159.1169(100), 173.1325(19), 157.1013(13), 145.1012(21), 119.0856(13)	Dimethyl-methylidene-hexahydrocyclodecafuranone isomer
54	9.72	229.12230	229.12202	−1.250	C_15_H_16_O_2_		MS[229]: 211.1117(15), 168.0933(28), 201.1272(11), 183.1168(100), 157.1012(19), 155.0854(20)	Methyl-dimethylidene-tetrahydroazulenofuranone isomer
55	9.77	211.11174	211.11154	−0.955	C_15_H_14_O		MS[211]: 196.0885(20), 168.0934(47), 193.1013(59), 183.1169(85), 181.1012(46), 157.1011(39), 155.0857(39)	Annulenone
56	10.07	305.24750	305.24728	−0.743	C_20_H_32_O_2_		MS[305]: 147.1167(58), 121.1014(56), 95.0859(100), 165.1278(22), 161.1324(38), 109.1015(87)	Yucalexin P21
57	10.09	303.23185	303.23163	−0.748	C_20_H_30_O_2_		MS[303]: 285.2210(67), 267.2170(44), 133.1013(58), 107.0858(53), 91.0861(62), 227.1793(100)	Hydroxy-dimethyl-methylidenetetracyclohexadecane carbaldehyde isomer
58	10.15	149.09609	149.09605	−0.279	C_10_H_12_O		MS[149]: 121.0650(100), 121.1013(12), 93.0703(3), 109.0651(4)	Tetrahydronaphthol isomer
59	10.22	345.13436	345.13474	4.274	C_19_H_22_O_6_	MS[345]: 201.1125(1), 115.0026(100), 97.9998(4), 99.0075(5), 71.0125(25)		Methyl-methylidene-(methylpropenoyloxy)-oxo-hexahydrocyclodecafuran carboxylic acid
60	10.24	347.18530	347.18481	−1.413	C_20_H_26_O_5_		MS[347]: 285.1848(100), 267.1744(69), 239.1794(12), 283.1694(27), 237.1272(14)	Gibberellin candidate (C_20_H_26_O_5_)
61	10.30	201.11323	201.11272	−2.536	C_10_H_18_O_4_	MS[201]: 139.11185(100), 181.10191(31)		sebacic acid
62	10.37	233.15360	233.15340	−0.885	C_15_H_20_O_2_		MS[233]: 135.08133(1), 159.11682(100), 177.12808(1), 215.14308(7)	Alantolactone
63	10.38	251.16417	251.16389	−1.119	C_15_H_22_O_3_		MS[251]: 233.1536(29), 187.1481(29), 159.1168(77), 119.0857(100), 175.1118(37), 147.1168(65), 205.1591(12)	Dihydrotomentosin
64	10.65	249.14852	249.14824	−1.128	C_15_H_20_O_3_		MS[249]: 157.10126(57), 159.11676(28), 175.11160(45), 185.13243(100), 231.13750(51)	Hydroxyalantolactone
65	10.69	347.15001	347.15030	3.990	C_19_H_24_O_6_	MS[347]: 329.1397(27), 287.0925(4), 285.1501(22), 241.1602(9), 161.0236(7)		Gibberellin candidate (C_19_H_24_O_6_)
66	10.80	211.11174	211.11156	−0.860	C_15_H_14_O		MS[211]: 196.0881(16), 168.0933(59), 183.1168(79), 157.1012(56), 155.0855(29), 193.1013(30)	Dimethylbiphenyl carboxaldehyde isomer
67	10.80	229.12230	229.12221	−0.420	C_15_H_16_O_2_		MS[229]: 168.0934(34), 183.1168(100), 157.1012(20), 141.0697(8), 131.0858(7)	Dimethyl-tetrahydrobenzocycloheptafuranone isomer
68	10.89	285.22129	285.22104	−0.884	C_20_H_28_O		MS[285]: 145.1012(100), 109.1013(29), 107.0858(26), 159.1167(26), 121.1015(29), 105.0701(29), 95.0859(39)	Dimethyl-(trimethylcyclohexenyl)-nonatrienynol isomer
69	11.11	233.15360	233.15341	−0.842	C_15_H_20_O_2_		MS[233]: 159.1168(100), 187.1482(22), 131.0856(13), 145.1012(15), 117.0701(7)	Dimethyl-methylidene-hexahydrocyclodecafuranone isomer
70	11.17	235.16925	235.16901	−1.048	C_15_H_22_O_2_		MS[235]: 161.1327(100), 189.1643(13), 133.1012(18), 119.0857(24), 217.1590(18), 105.0703(31), 95.0859(17)	Hydroxyisopatchoulenone
71	11.21	265.17982	265.17960	−0.834	C_16_H_24_O_3_		MS[265]: 233.1535(35), 187.1483(78), 159.1169(100), 145.1011(44), 107.0859(20), 119.0857(24)	Methyl hydroxy-dimethyl-hexahydronaphthalenylpropenoate isomer
72	11.21	245.11722	245.11703	−0.779	C_15_H_16_O_3_		MS[245]: 199.1118(100), 181.1012(24), 184.0884(11), 166.0780(9), 171.1168(13)	Dimethyl-methylidene-tetrahydroazulenofurandione isomer
73	11.22	251.16417	251.16396	−0.840	C_15_H_22_O_3_		MS[251]: 145.1013(49), 119.0857(100), 205.1589(59), 187.1481(73), 159.1167(97), 133.1012(45), 107.0859(40), 105.0702(40), 233.1534(36)	Hydroxy-dimethyl-hexahydronaphthalenylpropenoic acid isomer
74	11.57	233.15360	233.15343	−0.757	C_15_H_20_O_2_		MS[233]: 159.1168(100), 187.1428(23), 157.1010(12), 145.1012(17), 131.0857(10)	Dimethyl-methylidene-octahydroazulenofuranone isomer
75	11.79	345.13436	345.13483	4.535	C_19_H_22_O_6_	MS[345]: 327.1245(16), 285.0775(7), 229.0878(6), 283.1346(20)		Gibberellin
76	12.21	347.15001	347.15042	4.336	C_19_H_24_O_6_	MS[347]: 329.1404(30), 285.1499(24), 241.1601(9), 161.0239(5)		Gibberellin candidate (C_19_H_24_O_6_)
77	12.22	251.16417	251.16393	−0.960	C_15_H_22_O_3_		MS[251]: 215.1429(65), 187.1482(100), 161.1324(52), 159.1169(59), 135.1168(33), 147.1167(91), 145.1012(99), 131.0857(83)	Hydroxy-dimethyl-hexahydronaphthalenylpropenoic acid isomer
78	12.22	233.15360	233.15341	−0.842	C_15_H_20_O_2_		MS[233]: 215.1431(38), 187.1481(100), 159.1168(56), 161.1324(51), 145.1012(64), 135.1169(11)	Dimethyl-methylidene-hexahydrocyclodecafuranone isomer
79	12.35	253.17982	253.17958	−0.952	C_15_H_24_O_3_		MS[253]: 235.1690(30), 179.1074(24), 133.1013(100), 119.0856(73), 175.1483(60)	2,6-Dimethylidene-9-prop-1-en-2-ylcyclodecane-1,3,5-triol
80	12.43	449.28976	449.28940	−0.813	C_26_H_40_O_6_		MS[449]: 269.2260(60), 229.1950(100), 241.1946(14), 227.1791(13), 287.2373(18), 111.0807(32), 127.0391(33), 85.0290(87), 69.0343(16)	Retinyl glucoside
81	12.50	347.15001	347.15036	4.163	C_19_H_24_O_6_	MS[347]: 329.1397(20), 285.1499(17), 241.1596(11)		Gibberellin candidate (C_19_H_24_O_6_)
82	12.52	347.18530	347.18491	−1.125	C_20_H_26_O_5_		MS[347]: 283.1696(20), 285.1847(100), 267.1743(62), 109.1016(11), 107.0858(11), 119.0857(10)	Gibberellin candidate (C_20_H_26_O_5_)
83	12.52	329.17473	329.17441	−0.990	C_20_H_24_O_4_		MS[329]: 285.1849(45), 257.1743(100), 249.1643(3), 225.1278(5)	Trimethylidene-octahydroazulenofuranone 2-methylbut-2-enoate isomer
84	12.80	345.13436	345.13489	4.709	C_19_H_22_O_6_	MS[345]: 327.1239(6), 283.1344(9), 229.1234(12), 239.1440(6)	MS[347]: 283.1337(65), 237.1274(94), 231.1372(74), 185.1321(83), 195.1168(75), 145.1012(100)	Gibberellin candidate (C_19_H_22_O_6_)
85	12.86	229.12230	229.12221	−0.420	C_15_H_16_O_2_		MS[229]: 211.1116(20), 168.0932(20), 183.1168(100), 157.1011(34), 155.0854(25)	Dimethyl-methylidene-tetrahydroazulenofuranone isomer
86	12.89	183.11682	183.11670	−0.694	C_14_H_14_		MS[183]: 168.0933(57), 155.0859(51), 154.0781(9), 153.0696(20), 141.0698(17)	1,2-Bis(but-2-ynyl)benzene
87	12.90	285.22129	285.22095	−1.199	C_20_H_28_O		MS[285]: 185.1325(92), 211.1479(26), 145.1012(100), 109.1015(40), 93.0703(30), 107.0859(33), 81.0705(30)	11,12-Didehydro Retinol
88	12.97	347.15001	347.15039	4.249	C_19_H_24_O_6_	MS[347]: 329.1399(10), 285.1502(8), 115.0026(100)		Gibberellin candidate (C_19_H_24_O_6_)
89	13.08	305.24750	305.24719	−1.038	C_20_H_32_O_2_		MS[305]: 149.1327(39), 123.1169(52), 147.1166(41), 139.1120(39), 121.1012(64), 95.0860(90), 135.1168(88), 109.1016(100)	Torulosal
90	13.12	345.13436	345.13477	4.361	C_19_H_22_O_6_	MS[345]: 327.1243(8), 283.1344(12), 229.1233(10), 239.1438(5)		Gibberellin candidate (C_19_H_22_O_6_)
91	13.12	239.20055	239.20032	−0.989	C_15_H_26_O_2_		MS[239]: 147.1165(54), 121.1013(71), 95.0859(100), 161.1324(38), 135.1167(37), 123.1172(36)	7-Methyl-3-methylidene-10-propan-2-ylcyclodec-6-ene-1,5-diol
92	13.17	347.14891	347.14868	−0.677	C_19_H_22_O_6_		MS[347]: 237.1277(66), 211.1117(100), 265.1223(25), 267.1736(53), 231.1378(42), 213.1275(26), 99.0078(28)	Acetoxy-acetoxymethyl-dimethylidene-hexahydroazulenofuranone isomer
93	13.25	347.15001	347.15048	4.508	C_19_H_24_O_6_	MS[347]: 329.1398(23), 115.0026(100), 285.1500(19), 99.0075(9), 71.0125(25)		Gibberellin candidate (C_19_H_24_O_6_)
94	13.60	301.21620	301.21591	−0.985	C_20_H_28_O_2_		MS[301]: 255.2105(100), 227.1797(16), 161.1324(16), 145.1011(31), 119.0857(41), 107.0857(32)	Ethenyl-dimethyltetracyclopentadecene carboxylic acid isomer
95	13.61	309.16965	309.16928	−1.198	C_17_H_24_O_5_		MS[309]: 185.13255(100), 231.13799(43), 203.14294(55), 143.08562(51), 291.19482(7)	1-O-Acetyl Involucrasin
96	13.85	781.36654	781.36682	1.754	C_40_H_54_N_4_O_12_	MS[781]: 203.0555(5), 149.0451(4), 131.0342(7), 101.0596(60), 113.0232(28), 85.0282(10), 59.0126(100)		Bis[methyl(methylcarbamoyl) propyl]-bis[(methoxycarbonyl) ethenylbenzyloxy]-dihydroxyhexanediamide isomers
97	13.91	625.32185	625.32153	−0.517	C_32_H_48_O_12_		MS[625]: 255.2106(100), 283.2063(10), 147.1168(27), 121.1014(18), 145.1013(12), 119.0855(8), 103.0393(21), 85.0289(18), 109.1014(25), 159.1168(14)	Furanethyl-trimethyl-hexahydronaphthalene carboxylate hexose–deoxyhexose conjugate isomer
98	14.07	705.27529	705.27814	4.031	C_35_H_44_O_15_		MS[705]: 255.2105(100), 283.2055(18), 199.1480(15), 147.1167(26), 121.1013(21), 107.0858(15), 85.0289(13)	Methyl diacetoxy-dihydroxy-dimethyl-(2-methylbut-2-enoyloxy)-trioxatetracyclododecenyl-dioxatetracyclopentadecane carboxylate isomer
99	14.13	681.32535	681.32739	4.604	C_34_H_46_N_6_O_9_	MS[681]: 161.0447(46), 143.0336(23), 101.0232(100), 73.0281(25), 89.0231(69), 71.0124(44)		Cyclo[Gly-Thr-Phe-Leu-Tyr-Thr]
100	14.16	467.30033	467.29993	−0.856	C_26_H_42_O_7_		MS[467]: 305.2474(100), 287.2367(57), 269.2261(64), 257.2254(53), 153.1275(85), 135.1168(72), 109.1016(64)	Virescenoside Q
101	14.26	301.21620	301.21588	−1.085	C_20_H_28_O_2_		MS[301]: 255.2105(100), 199.1481(16), 175.1480(26), 95.0860(33), 161.1325(19), 135.1170(20)	Trimethyltetracyclohexadecadiene carboxylic acid isomer
102	14.32	319.22677	319.22638	−1.226	C_20_H_30_O_3_		MS[319]: 283.2050(22), 273.2212(65), 255.2104(100), 109.1015(58), 121.1012(47), 95.0860(40), 147.1168(35)	Steviol
103	14.76	301.21620	301.21591	−0.985	C_20_H_28_O_2_		MS[301]: 255.2106(100), 105.0703(93), 95.0860(69), 93.0704(40), 145.1011(85), 147.1169(51)	Grandiflorenic acid
104	15.00	327.21769	327.21811	4.613	C_18_H_32_O_5_	MS[327]: 229.1445(32), 211.1335(42), 171.1019(100), 221.1179(8), 181.1384(7), 137.0962(12)		Malyngic acid
105	15.24	329.17473	329.17419	−1.658	C_20_H_24_O_4_		MS[329]: 285.1847(30), 167.1742(100), 149.1630(5), 239.1804(6), 239.1424(11), 211.1479(9)	Cembratetraene diolide
106	15.25	677.28281	677.28021	−2.229	C_35_H_42_N_4_O_10_	MS[677]: 143.0703(25), 115.0753(100), 125.0598(16), 101.0596(53), 59.0125(21), 71.0125(17), 101.0232(13)		Hv-NCC-1
107	15.25	327.21769	327.21802	4.338	C_18_H_32_O_5_	MS[327]: 229.1444(61), 211.1337(100), 183.1384(60), 137.0964(7), 85.0282(25), 171.1020(55)		Trihydroxyoctadecadienoic acid isomer
108	15.26	275.20055	275.20023	−1.187	C_18_H_26_O_2_		MS[275]: 133.1013(100), 107.0858(36), 91.0547(40), 81.0705(38), 147.1169(58), 105.0703(76)	Octadecapentaenoic acid isomer
109	15.30	211.11174	211.11147	−1.287	C_15_H_14_O		MS[211]: 193.1012(79), 183.1168(100), 181.1011(80), 157.1012(63), 155.0855(44), 168.0932(54)	Cyclopentadecaheptaenone isomer
110	15.30	229.12230	229.12221	−0.420	C_15_H_16_O_2_		MS[229]: 211.1116(23), 168.0934(23), 183.1168(100), 157.1012(33), 155.0854(27)	Gweicurculactone
111	15.30	293.21112	293.21066	−1.573	C_18_H_28_O_3_		MS[293]: 275.2005(48), 133.1011(54), 135.0801(15), 109.0653(29), 81.0705(100), 107.0858(86), 97.1016(31)	Kot isomers
112	15.33	303.23185	303.23157	−0.946	C_20_H_30_O_2_		MS[303]: 99.0809(43), 95.0860(39), 71.0862(75), 121.0650(100), 161.0960(42), 109.1015(40)	Ethenyl-trimethyl-octahydrophenanthrene carboxylic acid isomer
113	15.33	183.11682	183.11673	−0.530	C_14_H_14_		MS[183]: 168.0933(56), 155.0855(46), 141.0689(22), 153.0700(18)	Bis(but-1-ynyl)benzene
114	15.42	295.22677	295.22644	−1.122	C_18_H_30_O_3_		MS[295]: 121.1013(89), 107.0859(59), 93.0704(82), 67.0550(65), 109.1016(38), 125.0964(24), 149.1327(19)	Hydroxylinolenic acid
115	15.45	345.15439	345.15411	−0.823	C_16_H_24_O_8_		MS[345]: 127.0391(55), 109.0287(44), 99.0808(38), 71.0862(100)	Bis(2-methylbut-2-enoyl)hexose isomer
116	15.55	327.21769	327.21808	4.521	C_18_H_32_O_5_	MS[327]: 291.1971(10), 197.1177(17), 171.1020(100), 211.1338(62), 183.1384(32), 229.1447(39)		Trihydroxyoctadeca-dienoic acid
117	15.56	293.21112	293.21082	−1.027	C_18_H_28_O_3_		MS[293]: 275.2010(58), 133.1014(65), 107.0858(74), 81.0705(100), 95.0860(90), 97.1015(34), 95.0496(36)	Kot isomers
118	15.61	211.11174	211.11154	−0.955	C_15_H_14_O		MS[211]: 193.1013(76), 183.1169(100), 181.1011(84), 155.0856(44), 157.1014(64), 168.0933(49), 196.0883(33)	Bis(2-methylphenyl)methanone
119	15.61	229.12230	229.12213	−0.770	C_15_H_16_O_2_		MS[229]: 211.1118(21), 168.0933(23), 183.1169(100), 157.1012(37), 155.0855(26)	Trimethylidene-hexahydroazulenofuranone isomer
120	15.61	183.11682	183.11671	−0.639	C_14_H_14_		MS[183]: 183.1168(100), 168.0933(53), 155.0854(48), 141.0697(19)	(14)Annulene
121	15.71	305.24750	305.24722	−0.940	C_20_H_32_O_2_		MS[305]: 153.1275(78), 135.1169(99), 109.1015(100), 139.1121(76), 121.1013(68), 95.0861(95), 59.0499(77), 147.1168(66)	Agatholal
122	15.77	781.36654	781.36676	1.677	C_40_H_54_N_4_O_12_	MS[781]: 149.0448(8), 131.0340(11), 113.0233(20), 85.0281(10), 59.0126(100)		Bis[methyl(methylcarbamoyl) propyl]-bis[(methoxycarbonyl) ethenylbenzyloxy]-dihydroxyhexanediamide isomer
123	15.94	327.21769	327.21808	4.521	C_18_H_32_O_5_	MS[327]: 229.1446(56), 211.1338(100), 183.1385(96), 171.1020(90), 225.1496(13), 197.1177(21)		Trihydroxyoctadeca-dienoic acid
124	16.01	305.24750	305.24719	−1.038	C_20_H_32_O_2_		MS[305]: 153.1273(66), 139.1118(69), 135.1169(88), 109.1017(89), 59.0499(62), 123.1171(52), 95.0860(100), 139.1117(69), 165.1274(50), 121.1014(56)	Hydroxykauran-18-al
125	16.01	467.30033	467.30005	−0.599	C_26_H_42_O_7_		MS[467]: 305.2470(100), 287.2362(51), 269.2269(57), 257.2261(37), 153.1272(90), 135.1170(66), 109.1014(51)	Hydroxy-dimethyl-methylidenetetracyclohexadecylmethyl hexoside isomer
126	16.03	599.34258	599.34216	−0.715	C_31_H_50_O_11_		MS[599]: 305.2473(100), 287.2373(32), 269.2253(25), 257.2262(20), 97.0289(35), 165.1275(26), 85.0289(30)	Ethenyl-tetramethyl-octahydrophenanthrene hexose/pentose diglycoside isomer
127	16.04	615.33993	615.33893	0.145	C_32_H_48_N_4_O_8_	MS[615]: 161.0447(19), 89.0232(100), 71.0126(95), 131.0340(25), 113.0232(48)		Dimethylaminoethylamino-hydroxy-methoxy-methoxymethyl-tetramethyl-trioxo-azabicyclohenicosapentaene
128	16.10	781.36654	781.36646	1.293	C_40_H_54_N_4_O_12_	MS[781]: 149.0449(10), 131.0341(6), 113.0233(26), 85.0282(12), 59.0126(100)		Bis [methyl (methylcarbamoyl) propyl ] -bis (methoxycarbonylethenylbenzyloxy)-dihydroxyhexanediamide isomer
129	16.52	611.34258	611.34247	−0.194	C_32_H_50_O_11_		MS[611]: 269.2261(100), 257.2251(12), 229.1951(12), 147.1168(68), 121.1014(48), 95.0859(66), 109.1015(65)	Furanethyl-tetramethyl-hexahydronaphthalenyl hexosyl-deoxyhexoside isomer
130	16.86	301.21620	301.21588	−1.085	C_20_H_28_O_2_		MS[301]: 255.2105(100), 199.1481(27), 145.1011(32), 119.0857(49), 147.1168(36), 107.0858(33)	Dimethyl-methylidenetetracyclohexadecene carboxylic acid isomer
131	16.94	329.23334	329.23352	3.856	C_18_H_34_O_5_	MS[329]: 229.1444(63), 211.1336(100), 171.1019(36), 183.1385(13), 139.1118(15), 99.0802(12)		Pinellic acid
132	16.94	327.21769	327.21790	3.971	C_18_H_32_O_5_	MS[327]: 197.1179(100), 171.1020(48), 211.1336(43), 201.1126(23), 185.1179(37), 183.1384(62), 209.1180(39)		dihydroxy-oxooctadec-enoic acid
133	17.16	345.08162	345.08099	−1.835	C_14_H_16_O_10_		MS[345]: 75.0863(100), 125.09563(3), 169.05003(4)	Vanillic acid glucuronide
134	17.19	303.23185	303.23160	−0.847	C_20_H_30_O_2_		MS[303]: 257.2262(100), 175.1484(14), 149.1326(21), 95.0860(22), 161.1324(19), 81.0704(15)	Pimaric Acid
135	17.27	891.47475	891.47632	2.981	C_47_H_72_O_16_	MS[891]: 247.1340(100), 203.1438(12), 178.0630(5), 185.1328(3)		Soyasaponin gamma-A
136	17.27	301.21620	301.21591	−0.985	C_20_H_28_O_2_		MS[301]: 255.2102(100), 283.2056(23), 133.1012(57), 121.1014(54), 95.0858(60), 145.1010(33), 147.1170(73)	Dimethyl-methylidenetetracyclohexadecene carboxylic acid isomer
137	17.29	319.22677	319.22635	−1.320	C_20_H_30_O_3_		MS[319]: 301.2159(31), 273.2210(76), 255.2105(100), 121.1015(72), 95.0859(60), 59.0500(48), 109.1016(72)	Grandifloric acid
138	17.31	329.23334	329.23364	4.220	C_18_H_34_O_5_	MS[329]: 229.1444(55), 211(82), 171.1020(100), 183.1386(13), 139.1119(37), 127.1117(13)		Trihydroxy-octadecenoic acid
139	17.34	327.21769	327.21805	4.430	C_18_H_32_O_5_	MS[327]: 239.1652(43), 209.1179(100), 225.1493(41), 197.1179(59), 171.1020(57)		Dihydroxy-oxooctadec-enoic acid
140	17.74	289.25259	289.25232	−0.941	C_20_H_32_O		MS[289]: 271.2418(83), 175.1483), 161.1327(47), 109.1015(84), 95.0860(98), 81.0705(100)	Dihydroretinol
141	18.14	293.21112	293.21069	−1.471	C_18_H_28_O_3_		MS[293]: 133.1010(27), 125.0964(26), 107.0858(100), 95.0494(28), 91.0546(22), 81.0705(38), 97.1016(29), 147.1169(25)	Hydroxyoctadeca-dien-ynoic acid
142	18.15	329.23334	329.23389	4.979	C_18_H_34_O_5_	MS[329]: 293.2131(26), 211.1337(35), 201.1129(93), 171.1020(87), 199.1337(34)		Tianshic acid
143	18.15	327.21769	327.21826	5.071	C_18_H_32_O_5_	MS[327]: 291.1975(40), 197.1179(100), 213.1130(41), 185.1177(96), 201.1127(70)		Trihydroxyoctadeca-dienoic acid
144	18.36	293.21112	293.21072	−1.369	C_18_H_28_O_3_		MS[293]: 107.0859(49), 91.0546(34), 81.0705(100), 121.1014(33), 95.0860(82), 69.0706(78), 105.0703(37)	Kot isomers
145	18.53	329.23334	329.23389	4.797	C_18_H_34_O_5_	MS[329]: 201.1128(76), 171.1019(100), 155.1068(8), 199.1337(20), 127.1117(10)		Sanleng acid
146	18.54	305.24750	305.24716	−1.136	C_20_H_32_O_2_		MS[305]: 139.1120(58), 121.1013(53), 95.0860(100), 59.0500(65), 135.1171(96), 109.1014(91), 93.0705(35)	Ent-Hydroxy-kauran-al
147	18.73	289.25259	289.25229	−1.045	C_20_H_32_O		MS[289]: 271.2418(67), 161.1326(54), 135.1169(59), 109.1015(77), 149.1326(55), 95.0860(100)	(Trimethyl-tetracyclo-hexadec-enyl)methanol
148	18.83	303.23185	303.23151	−1.143	C_20_H_30_O_2_		MS[303]: 257.2263(100), 201.1633(11), 121.1014(39), 95.0860(29), 81.0705(27), 175.1481(36), 149.1325(53)	Isoozic acid
149	19.08	293.21112	293.21088	−0.241	C_18_H_28_O_3_		MS[293]: 149.0962(100), 121.1014(42), 107.0459(85), 105.0703(62), 95.0860(51), 95.0496(97), 93.0703(65)	Kot isomers
150	19.15	735.32334	735.32495	3.676	C_37_H_52_O_15_	MS[735]: 301.1813(100), 101.0596(11), 59.0125(32)		Triacetoxy-bis(3-methylbutanoyloxy)-hydroxy-methoxymethyl-dimethyl-oxo-spiro(oxatricycloheptadecadiene-oxirane)
151	19.16	301.21620	301.21594	−0.885	C_20_H_28_O_2_		MS[301]: 255.2106(100), 105.0702(26), 95.0859(22), 145.1013(36), 119.0857(52), 109.1015(34), 107.0859(32)	Trimethylpentacyclo-hexadec-ene-carboxylic acid
152	19.26	329.23334	329.23376	4.585	C_18_H_34_O_5_	MS[329]: 311.2229(18), 293.2126(15), 211.1336(40), 201.1128(37), 171.1020(37), 169.1228(13)		Trihydroxy-octadecenoic acid
153	19.39	303.23185	303.23154	−1.045	C_20_H_30_O_2_		MS[303]: 257.2263(100), 201.1637(15), 149.1325(29), 95.0860(32), 109.1015(17)	Isopimaric Acid
154	19.74	289.25259	289.25226	−1.148	C_20_H_32_O		MS[289]: 271.2422(86), 189.1637(75), 161.1327(43), 163.1482(32), 149.1325(77), 109.1015(97), 95.0860(100)	Trimethyl-methylidene-hexahydronaphthalenyl-methylpentenal
155	20.15	293.21112	293.21075	−1.266	C_18_H_28_O_3_		MS[293]: 275.2004(100), 107.0859(78), 81.0705(72), 121.1014(49), 95.0859(43), 93.0703(70)	Kot isomers
156	20.38	303.23185	303.23141	−1.473	C_20_H_30_O_2_		MS[303]: 257.2262(100), 121.1013(41), 175.1482(34), 95.0860(27), 135.1169(34)	Kaurenoic Acid
157	20.39	761.29992	761.29956	0.964	C_34_H_46_N_6_O_14_	MS[761]: 485.2526(57), 401.2704(58), 361.2393(60), 101.0232(74), 57.0333(100)		Aminomethylfuran-methoxyphenethyl tetraglutamyl conjugate
158	20.68	293.21112	293.21082	−1.027	C_18_H_28_O_3_		MS[293]: 275.2003(100), 133.1012(65), 81.0705(56), 91.0548(30), 105.0703(61), 69.0706(48)	Kot isomers
159	21.03	311.22278	311.22345	5.668	C_18_H_32_O_4_	MS[311]: 293.2126(52), 225.1494(20), 211.1338(100), 171.1020(96), 181.1231(27), 199.1338(25)		Filoboletic acid
160	22.68	295.22786	295.22806	4.365	C_18_H_32_O_3_	MS[295]: 277.2176(100), 195.1386(38), 171.1021(50), 183.1026(5), 179.1442(3), 183.1392(4)		Hode
161	24.54	331.28428	331.28381	−1.437	C_19_H_38_O_4_		MS[331]: 109.1015(23), 85.1017(52), 83.0861(25), 67.0549(17), 57.0707(100), 71.0862(79), 95.0860(52)	Monopalmitin
162	25.37	359.31558	359.31522	−1.019	C_21_H_42_O_4_		MS[359]: 97.1016(21), 71.0862(80), 57.0707(100), 95.0860(54), 85.1017(50), 109.1015(26)	Monostearin

Note: All data correspond to compounds supported by accurate mass spectrometry, isotopic patterns, MS/MS fragmentation, FBMN relationships, SIRIUS candidate rankings, and database/literature comparisons, and are currently classified as level 3 tentatively annotated.

## Data Availability

The original contributions presented in this study are included in the article. Further inquiries can be directed to the corresponding authors.
